# Effect of Solvent Properties on the Critical Solution Temperature of Thermoresponsive Polymers

**DOI:** 10.3390/ijms25147734

**Published:** 2024-07-15

**Authors:** Konstantin Nikolaus Beitl, Erik Reimhult

**Affiliations:** Institute of Colloid and Biointerface Science, Department of Bionanosciences, BOKU University, Muthgasse 11, A-1190 Vienna, Austria

**Keywords:** thermoresponsive polymers, critical solution temperature, Hofmeister series anions

## Abstract

The ability of thermoresponsive polymers to respond to temperature with a reversible conformational change makes them promising ‘smart’ materials for solutions in medical and biotechnological applications. In this work, two such polymers and structural isomers were studied: poly(*N*-isopropyl acrylamide) (PNiPAm) and poly(2-isopropyl-2-oxazoline) (PiPOx). We compare the critical solution temperatures (CST) of these polymers in D_2_O and H_2_O in the presence of Hofmeister series salts, as results obtained under these different solvent conditions are often compared. D_2_O has a higher dipole moment and electronegativity than H_2_O, which could significantly alter the CST transition. We used two complementary methods to measure the CST, dynamic light scattering (DLS) and differential scanning calorimetry (DSC) and found that the CST decreased significantly in D_2_O compared to H_2_O. In the presence of highly concentrated kosmotropes, the CST of both polymers decreased in both solvents. The influence of the kosmotropic anions was smaller than the water isotope effect at low ionic strengths but considerably higher at physiological ionic strengths. However, the Hofmeister anion effect was quantitatively different in H_2_O than in D_2_O, with the largest relative differences observed for Cl^−^, where the CSTs in D_2_O decreased more than in H_2_O measured by DLS but less by DSC. PiPOx was more sensitive than PNiPAm to the presence of chaotropes. It exhibited much higher transition enthalpies and multistep transitions, especially in aqueous solutions. Our results highlight that measurements of thermoresponsive polymer properties in D_2_O cannot be compared directly or quantitatively to application conditions or even measurements performed in H_2_O.

## 1. Introduction

The ability of a material to react to external stimuli and alter its shape and functionality is a crucial characteristic of so-called ’smart’ (bio)materials. In response to environmental cues like changes in pH, temperature, redox potential, or ionic strength, these structures can adapt their behavior or trigger desired reactions. This is an exciting property for novel engineering solutions in biotechnology and medicine [1,2,3]. One example of such a material that has gained more and more attention since its introduction in the late 1960s is thermoresponsive polymers [4,5,6]. Upon reaching a specific temperature, thermoresponsive polymers dispersed in a solvent change their conformation from an expanded, well-soluble state to a collapsed, poorly soluble globular state over a narrow temperature range [7]. This process is reversible. A thermoresponsive polymer can exhibit a lower or upper critical solution temperature (LCST or UCST) and thereby decrease or increase its solvation, respectively, when the temperature is raised past its critical solution temperature (CST). The CST is concentration- and solvent-dependent.

What makes thermoresponsive polymers especially interesting for researchers is that adaptive behavior adds multifunctionality, which makes thermoresponsive polymers promising for applications in, e.g., tissue engineering, drug delivery, or catalysis [8,9,10].

The exact CST or temperature range at which the transition happens depends on the polymer and the solvent environment. Solvent polarity or changes to pH or ion concentration alter the CST of thermoresponsive polymers. For instance, intermolecular interactions in bulk deuterium oxide (D_2_O) are stronger compared to water. D_2_O molecules bind more strongly to polar groups of, e.g., polymer chains, and the resulting entropic penalty of exposed non-polar segments to the polar solvent molecules is higher [11]. This is crucial because some essential analytical techniques rely on D_2_O as a measurement solvent. Prominent examples are nuclear magnetic resonance spectroscopy (NMR), small-angle neutron scattering (SANS), and neutron reflectometry (NR), which are powerful techniques for investigating polymer structures in bulk and at interfaces. However, applications are designed for biological fluids (H_2_O), where the polymer conformation and energetics could differ. A direct comparison of measurements with these techniques to complementary measurements by other techniques in normal water could be misleading.

Further, biological fluids contain high concentrations of Hofmeister series salts. The Hofmeister series describes salts according to their ability to affect the stability of proteins or polymers via their effect on the inter-water bonding network [12,13] and polar bonds of the polymer [14,15]. Historically, these species were often described as ‘structure-making’ or ‘structure-breaking’ compounds because of their effect on bulk water. Regarding the stability of proteins, kosmotropes are known to promote and stabilize their defined folded conformation. Chaotropes, on the other hand, lead to the denaturation of proteins [12,16]. More recent findings, notably contributed by the Cremer group, suggest that the actual mechanism behind these effects is connected to the relative ion polarizabilities and the specific ion interactions directly with the macromolecular solutes and their immediately adjacent hydration shells [14,15]. Moreover, the intermolecular interactions of H_2_O or D_2_O molecules differ when such solutes are added [17,18,19]. The influence of Hofmeister ions on the CST can be huge and vary depending on the polymer [20], which agrees with the results of Cremer et al.

Despite the use of D_2_O instead of H_2_O in crucial analytical techniques to study (bio)polymer conformations and their thermoresponsive properties, there is a lack of studies on the differences in the thermal solvation transition between them. Additionally, given the importance of the effect of Hofmeister ions on the solvent structure for polymer stability, there is a lack of data on how they affect structure in D_2_O. This is a severe deficiency of the current literature, given that much of the characterization of polymer properties is performed in D_2_O and not H_2_O and, further, that these investigations are performed in pure D_2_O without the presence of Hofmeister series ions. Still, such measurements are compared directly to other results obtained under physiological conditions in H_2_O to draw conclusions for designing applications. Additionally, a disproportionate amount of research on the thermal transition of polymers in water was performed on poly(*N*-isopropyl acrylamide) (PNiPAm). It is the most commonly investigated thermoresponsive polymer with applications in biotechnology [21,22,23]. It has, e.g., been used as a surface modification to create planar substrates for cultivating cell sheets, first demonstrated by Okano et al. in 1993 [24]. This success has sparked extensive research on various medical applications [25,26,27,28]. Other popular thermoresponsive polymers, e.g., poly(2-isopropyl-2-oxazoline) (PiPOx), are less thoroughly investigated with salts co-solved during measurements. PiPOx is a promising material for biomedical applications because PiPOx exhibits better biocompatibility than PNiPAm, reacts more strongly to environmental changes, and has a tunable CST closer to human body temperature [29,30]. 

Published research on thermoresponsive polymers like PNiPAm or PiPOx primarily covers specific cases, e.g., investigating these polymers crosslinked in hydrogels, grafted to surfaces, or as part of more complex copolymers [31,32,33]. Our study tries to fill the gap with data connecting the CST of PNiPAm and PiPOx to solvent and co-solute properties that include D_2_O. Two complementary experimental techniques proper for both D_2_O and H_2_O were applied to investigate the CST: dynamic light scattering (DLS) and differential scanning calorimetry (DSC). DLS and DSC are two of the most frequently used techniques for determining critical solution temperatures. These techniques provide complementary information about the investigated systems, as they measure the CST in fundamentally different ways. By including data collected with both methods in this work, we aim to paint a more complete picture of the transition of thermoresponsive polymers than is available in the existing literature [20,34,35].

## 2. Results

### 2.1. Effect of Polymer Concentration on the CST in H_2_O and D_2_O

CST values were extracted from DLS and DSC measurements for PNiPAm and PiPOx of 21,000 g mol^−1^, respectively. DLS determines particle size from fluctuations in the scattered light intensity. The total scattered light intensity increases when aggregates are formed, which can also be measured sensitively with a DLS instrument. Once the CST has been reached, aggregates are formed. They sediment due to gravity and move in the bulk medium due to thermal convection in addition to Brownian motion. As a result, the recorded light intensity does not monotonously increase as aggregation sets in; it is susceptible to fluctuations depending on the movement of large scattering objects within the sampling volume of the DLS. In this study, DLS-derived CST values were, therefore, extracted from the initial increase in the scattering intensity as a function of temperature. A graphical illustration of the selection is shown in Figure S1 in the Supplementary Information. The selected points correspond to the temperature at which the collapsing polymer coils detectably start aggregating in response to their thermally induced reduced solvation. In contrast, DSC measures the energetics of the coil-globule transition. We recorded the temperature at the transition peak maximum as the CST. Although this point does not represent the start of the transition, it facilitates comparing the stability of polymers, as was shown in other studies [36,37]. 

**Figure 1:**
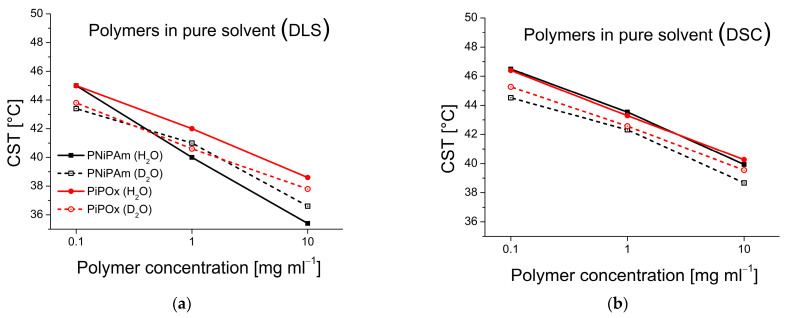
Effect of solvent and polymer concentration on the CST (PiPOx and PNiPAm: 21 kDa). Black lines: CST values of PNiPAm, red lines: CST values of PiPOx; solid lines: polymers dispersed in H_2_O, dashed lines: polymers dispersed in D_2_O. (**a**) Measurements with DLS; (**b**) measurements with DSC.

In Figure 1, CST values of differently concentrated PNiPAm and PiPOx samples in H_2_O and D_2_O are shown (see Table S1 in the Supplementary Material for exact values). Results from DSC and DLS experiments with PiPOx correspond well, as CST values follow the same trend of decreased temperatures with increasing polymer concentration. The CST determined by DLS is approximately 4 °C lower than for DSC for low (0.1 mg mL^−1^) polymer concentrations, which decreases to 1 °C for high (10 mg mL^−1^) concentrations. These values are close to the temperature difference between the peak onsets and peak maxima of the DSC thermograms. These also vary with concentration and are approximately 4 °C lower at 0.1 mg mL^−1^ and 1 °C lower at 10 mg mL^−1^ (see Table S2). The CST decreases monotonously and quasi-linearly in the investigated concentration regime in a semi-log plot. The CST changes as a function of concentration measured by DSC are very similar in the case of all polymer and solvent combinations. The CST drops approximately 5 °C from 0.1 to 10 mg mL^−1^. Measured by DLS, the CST drops by 5 °C for PiPOx but slightly more steeply for PNiPAm, with close to 10 °C in H_2_O and close to 7 °C in D_2_O, in the same concentration range. A decrease of approximately 3 °C for an increase in polymer concentration from 0.1 mg mL^−1^ to 1 mg mL^−1^ was reported by Uyama and Kobayashi in 1992 for PiPOx of comparable molecular weight ($M_{w}=18.9\;kDa$) [38]. Our results are in the same range as their observations, which they made visually using turbidimetry. Results reported by Pamies et al. show a CST shift of PNiPAm of about 4 °C between 0.1 and 1 mg mL^−1^, which corresponds well to our findings, considering they measured PNiPAm at a lower molecular weight ($M_{w}=13.4\;kDa$) [39].

Comparing H_2_O and D_2_O, the CSTs determined by DSC are lower in D_2_O for both PiPOx and PNiPAm, with, on average, 1.0 °C and 1.5 °C, respectively. The dependence of the difference as a function of concentration is moderate, with 0.72 °C difference for the 10 mg mL^−1^ and 1 mg mL^−1^ samples and 1.12 °C in the case of the 0.1 mg mL^−1^ sample. PiPOx shows a similar dependence on the solvent measured by DLS to that observed by DSC. The CST measured by DLS is approximately 1.0 °C lower in D_2_O. In contrast, PNiPAm has a higher CST in D_2_O than in H_2_O, as measured by DLS at higher concentrations. Only at 0.1 mg mL^−1^ is a higher CST in H_2_O observed. Other studies have reported higher CST values for PNiPAm in D_2_O as well, although the measured systems differ from ours: Xiaohui Wang and Chi Wu measured a 1.5 °C upwards shift of the CST in D_2_O for huge PNiPAm molecules in highly dilute dispersions (13,000 kDa, $6.3\times 10^{-4}\;mg\;mL^{-1}$) using a combination of static and dynamic light scattering methods [40]. Shirota et al. aimed to determine the CST of PNiPAm hydrogels by microscopically observing the volume phase transition and also reported a higher CST in D_2_O of approximately 1 °C [31]. We note that all these studies also relied on observing the aggregation of PNiPAm at high local concentrations rather than the solvation transition directly.

**Figure 2:**
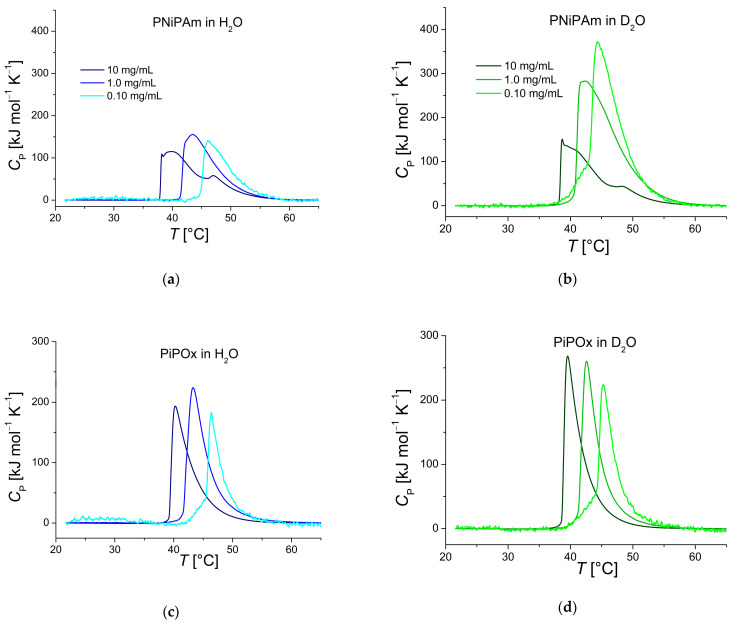
DSC recorded thermograms of PNiPAm and PiPOx (21 kDa) measured in (**a**,**c**) D_2_O and (**b**,**d**) H_2_O. Darker colors represent higher polymer concentrations.

Figure 2a shows DSC thermograms of PNiPAm in H_2_O and D_2_O. The long high-temperature tails hint at a more complex process than a simple two-state transition. In contrast, the PiPOx thermograms shown in Figure 2c,d suggest that the transitions differ less between the highest and lowest concentrations than for PNiPAm. In particular, the heating curves of 10 mg mL^−1^ PNiPAm plotted in panels (a) and (b) of Figure 2 show more than one peak after a very sharp initial incline.

### 2.2. Hofmeister Series Anions and Solvent Isotope Effect

**Figure 3:**
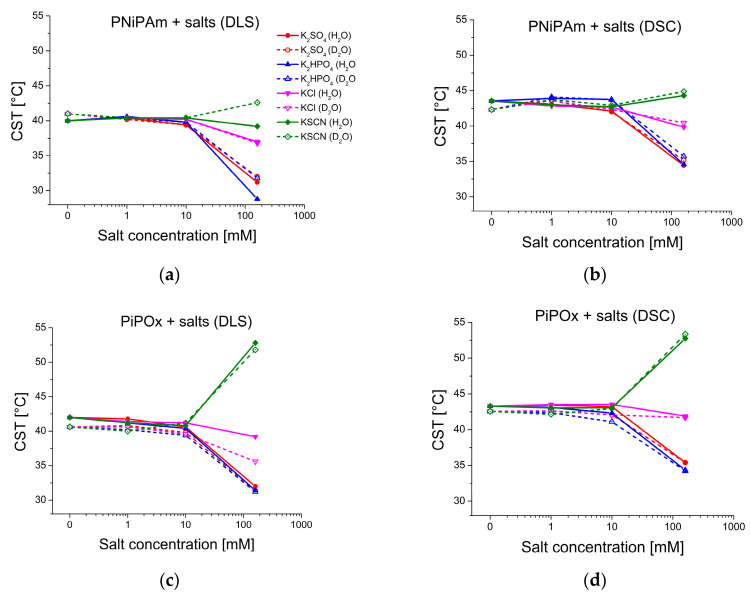
Effect of salt concentration (0 mM, 1 mM, 10 mM, and 160 mM) on polymer CST in H_2_O and D_2_O (PiPOx and PNiPAm: 21 kDa; 1 mg mL^−1^). Red: $K_{2}{\mathrm{SO}}_{4}$, blue: $K_{2}{\mathrm{HPO}}_{4}$, pink: KCl, green: KSCN; solid lines: measured in H_2_O, dashed lines: D_2_O. The concentrations are plotted on a logarithmic scale. (**a**) PNiPAm measured with DLS; (**b**) PNiPAm measured with DSC; (**c**) PiPOx measured with DLS; (**d**) PiPOx measured with DSC.

**Figure 4:**
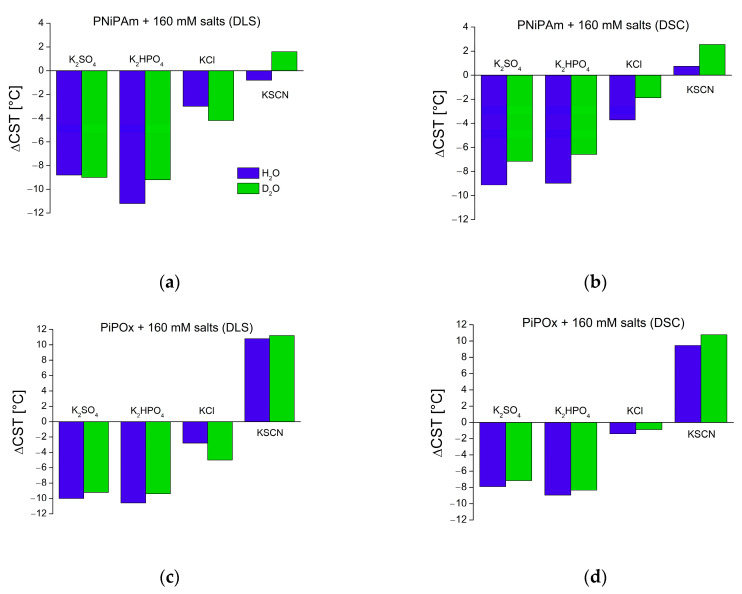
Effect of physiological salt concentration (160 mM) on polymer CST in H_2_O and D_2_O (PiPOx and PNiPAm: 21 kDa, 1 mg mL^−1^) compared to pure solvents. Blue: measured in H_2_O, green: D_2_O. (**a**) PNiPAm measured with DLS; (**b**) PNiPAm measured with DSC; (**c**) PiPOx measured with DLS; (**d**) PiPOx measured with DSC.

Figure 3 shows the CST values of both polymers derived from DLS and DSC experiments. The CST was measured in the pure solvents, as well as in the presence of differently concentrated potassium salts of four different Hofmeister series anions: thiocyanate (${\mathrm{SCN}}^{-}$), chloride (${\mathrm{Cl}}^{-}$), hydrogen phosphate (${\mathrm{HPO}}_{4}^{2^{-}}$), and sulfate (${\mathrm{SO}}_{4}^{2^{-}}$), representing chaotropic and kosmotropic anions of the Hofmeister series. Of these, ${\mathrm{SCN}}^{-}$ is a strong chaotrope, ${\mathrm{Cl}}^{-}$ a weak chaotrope, and ${\mathrm{HPO}}_{4}^{2^{-}}$ and ${\mathrm{SO}}_{4}^{2^{-}}$ strong kosmotropes [12]. It is noticeable that with increasing salt concentration, CSTs shift further away from the values in pure solvent (see also Table S3). This is true for both polymers in H_2_O and D_2_O, regardless of the measurement method. However, up to 10 mM concentration, all the salt effects are minor or negligible. For example, the CST value of PiPOx in 1 mM KSCN is the same as for 1 mM $K_{2}{\mathrm{HPO}}_{4}$ in H_2_O (both 41.2 °C) and even slightly lower in D_2_O (40.0 °C for 1mM KSCN compared to 40.2 °C of PiPOx in 1 mM $K_{2}{\mathrm{HPO}}_{4}$). The chaotropic properties of ${\mathrm{SCN}}^{-}$ are not visible at the lowest concentration. These results show that significant changes to the CST, as measured by both DLS and DSC, only occur for ionic strengths in the 100 mM range. However, DLS measurements of PNiPAm or PiPOx in solutions of physiological ionic strength (160 mM potassium salts) clearly show how chaotropic and kosmotropic substances from the Hofmeister series affect polymer stability. Figure 4 highlights these differences by comparing the CST of both polymers in a pure solvent and the presence of 160 mM salts. 

The kosmotropic anion concentration generally affects PiPOx in the same way in H_2_O and D_2_O. Figure 3 and Figure 4 show how high concentrations of strong kosmotropes like ${\mathrm{SO}}_{4}^{2^{-}}$ and ${\mathrm{HPO}}_{4}^{2^{-}}$ drastically reduce the CST of both polymers in pure H_2_O or D_2_O by approximately 7–10 °C. The chaotropic anion ${\mathrm{SCN}}^{-}$ promotes hydration of the polymer chain, increasing the CST. However, the effect is not as pronounced for PNiPAm as the effect of the kosmotropic ions. In the case of PNiPAm in H_2_O, low concentrations of KSCN do not increase the CST above the value measured in pure water. The same result is observed for PiPOx samples measured with both DLS and DSC for low ionic strengths, but the chaotropic effect of KSCN is on par with the kosmotropic ions at physiological ionic strength, shifting the CST higher by about 10 °C for PiPOx. 

Comparing DSC peak maxima, CST shifts between H_2_O and D_2_O are generally smaller than 1 °C for PiPOx in the presence of all salt concentrations (see Table S3). The same is true for CST values determined with DLS. The DSC measurements for PNiPAm are significantly different in 160 mM ionic strength, where the change in the CST values in D_2_O is lower than in H_2_O, generally with a difference in the shift of ~2 °C. Notably, the change of the CST for the chaotropic ion ${\mathrm{SCN}}^{-}$ is about 1.8 °C higher in D2O than in H_2_O, indicating that PNiPAm retains a higher hydration in H_2_O than in D_2_O. An outlier compared to the other ions is KCl, for which H_2_O seems to induce a lower effect on the hydration than D_2_O for both PNiPAm and PiPOx.

### 2.3. Transition Energetics

In Figure 2a,b, very large transition enthalpies per monomer unit are observed for PNiPAm (0.1 mg mL^−1^ and 1 mg mL^−1^) measured in D_2_O compared to in H_2_O. These samples require approximately twice the enthalpy per monomer unit compared to the same sample in H_2_O (11,838 J mol^−1^ and 11,459 J mol^−1^ versus 4178 J mol^−1^ and 5568 J mol^−1^; see Table 1). In comparison, Diab et al. reported a transition enthalpy of about 3347 J mol^−1^ for 1 mg mL^−1^ PNiPAm (13 kDa) in H_2_O [41], which is similar to our result for the same concentration. They did not, however, measure PNiPAm in D_2_O. PiPOx did not show such significant differences in transition enthalpy and had a more uniform peak shape (Figure 2c,d). In the same study, Diab et al. also measured transition enthalpies of PiPOx (5.7 kDa, 1 mg mL^−1^) of 5648 J mol^−1^ per monomer unit in H_2_O and 5983 J mol^−1^ in D_2_O. Even though the PiPOx measured in their experiments was almost four times smaller than ours, the measured transition enthalpies are in the same range. In our study, however, there seems to be a weak correlation of a moderate increase in transition enthalpy for both polymers in H_2_O. In D_2_O, there are only minor differences in transition enthalpy for PiPOx. Still, PNiPAm shows a dramatic difference in peak *C*_p_ and transition enthalpy for the lower concentrations compared to the highest. Both decrease with increasing concentration (Table 1 and Figure 2). 

**Figure 5:**
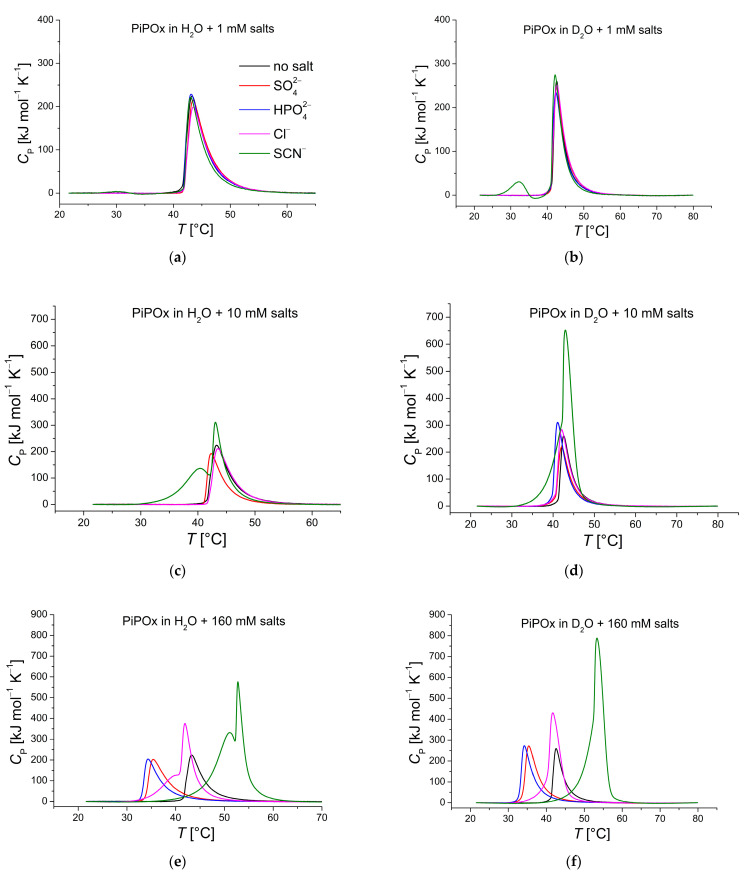
DSC recorded thermograms of PiPOx (21 kDa, 1 mg mL^−1^) in H_2_O and D_2_O. Red: $K_{2}{\mathrm{SO}}_{4}$, blue: $K_{2}{\mathrm{HPO}}_{4}$, pink: KCl, green: KSCN; polymer dispersed in solutions of (**a**) 1 mM salts in H_2_O; (**b**) 1 mM salts in D_2_O; (**c**) 10 mM salts in H_2_O; (**d**) 10 mM salts in D_2_O; (**e**) 160 mM salts in H_2_O; and (**f**) 160 mM salts in D_2_O.

Figure 5 presents thermograms of PiPOx in H_2_O and D_2_O co-solved with salts of increasing varying concentration. In contrast to PNiPAm, PiPOx is affected more profoundly in its transition in the presence of some salts (compare Figure 5 and Figure S2), as seen from the shoulders and a second peak. The most pronounced effects are observed for the strong chaotrope ${\mathrm{SCN}}^{-}$ and the weak chaotrope ${\mathrm{Cl}}^{-}$ in H_2_O. 

**Figure 6:**
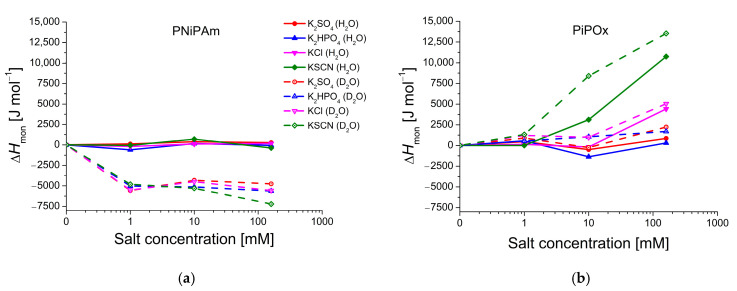
Effect of salt concentration (1 mM, 10 mM, and 160 mM) on polymer transition enthalpies per monomer unit (Δ*H*_mon_) in H_2_O and D_2_O (PiPOx and PNiPAm: 21 kDa; 1 mg mL^−1^). Red: $K_{2}{\mathrm{SO}}_{4}$, blue: $K_{2}{\mathrm{HPO}}_{4}$, pink: KCl, green: KSCN; solid lines: measured in H_2_O, dashed lines: D_2_O. Plotted are Δ*H*_mon_ values compared to *H*_mon_ in the pure solution of (**a**) PNiPAm and (**b**) PiPOx, as a function of the concentration plotted on a logarithmic scale.

Data presented in Figure 6 shows that the multi-step conformational change of PiPOx in the presence of medium to high concentrations of KSCN requires far more heat than in the presence of other salts, with $\Delta H_{mon}$ ranging from 7838 J mol^−1^ (PiPOx in a solution of 10 mM KSCN in H_2_O) to 18,000 J mol^−1^ (PiPOx in D_2_O + 160 mM KSCN), compared to other samples with transition enthalpies mostly between 4000 and 6000 J mol^−1^. This chaotrope thereby uniquely stabilizes the solvated polymer, as also evidenced by the higher CST. Although 160 mM KCl still has a kosmotropic effect on the CST, it produces a much higher transition enthalpy, especially in H_2_O; it displays pronounced double-peaks and transition enthalpies of 9135 J mol^−1^ in H_2_O and 9514 J mol^−1^ in D_2_O at 160 mM (Figure 5e,f). In the PNiPAm samples, there seems to be no connection between CST and transition enthalpy. Values for $\Delta H_{mon}$ vary by approximately only 12% between samples of the same salt concentration in H_2_O and D_2_O, except for PNiPAm in pure D_2_O, for which it is almost twice as high as in H_2_O or in D_2_O salt solutions (11,459 J mol^−1^; Figure 6 and Table S4).

## 3. Discussion

Even though PNiPAm and PiPOx are structural isomers, they did not respond in the same way to being solvated in D_2_O instead of H_2_O. PiPOx clearly decreases its CST in D_2_O. D_2_O seems to decrease the CST for PNiPAm as well, at least when the polymer is solvated at a low concentration, but the DSC measurements reveal a possible two-step transition that becomes more accentuated in D_2_O and leads to a strong concentration dependence (Figure 2). Hence, the CST determined from DLS is higher in D_2_O than in H_2_O, while the CST determined from the DSC peak of the main transition is lower in D_2_O than in H_2_O. Previously published literature does not report similar features in the DSC curves, but experimental conditions differ from the measurements conducted in this study. For instance, Qiu et al., did not observe these features for PNiPAm of molecular weights between 7.6 and 44.5 kDa, and with other end-groups like hydroxyethyl, *n*-butyl, or isobutylsulfanylthiosulfanyl [42]. To investigate this phenomenon in more detail, follow-up research would be required, especially to pinpoint if the switch from H_2_O to D_2_O influences the hydration of different hydrogen-bonding groups on the polymers differently. 

Figure 2 also reveals that the phase transition is sharper for PiPOx in both H_2_O and D_2_O, especially at a high polymer concentration (10 mg mL^−1^). An in-depth study of the demixing and remixing kinetics of aqueous PiPOx solutions conducted by Zhao et al. in 2010 attributes this to a faster thermal response of PiPOx compared to PNiPAm [43]. This can be explained by PiPOx forming relatively weaker hydrogen bonds than PNiPAm in solution, as it lacks amide groups in its side chain.

Compared to the Hofmeister series ions’ effects on the CST of PNiPAm and PiPOx, we observe that D_2_O has a relatively small influence. Notably, 160 mM strong kosmotropes and chaotropes solutions influenced the CSTs and the energetics of the solubility transition the most. Still, the difference between H_2_O and D_2_O was on par or larger than the effect of Hofmeister salts up to salt concentrations of 10 mM, especially for PiPOx. Both polymers significantly decrease their CST in response to strong kosmotropes (${\mathrm{SO}}_{4}^{2^{-}}$ and ${\mathrm{HPO}}_{4}^{2^{-}}$) in D_2_O and H_2_O. 

Interestingly, the relative effect of D_2_O on the CST (in particular measured by DLS) and the transition energy were most pronounced for ${\mathrm{SCN}}^{-}$ and ${\mathrm{Cl}}^{-}$, which are usually classified as strong and weak chaotropes, respectively. A first explanation could be that anions like ${\mathrm{SCN}}^{-}$ are weaker hydrated in D_2_O than water and bind closer to the polymer chain. This could lead to enhanced stabilization, i.e., a higher CST [11,44]. Hence, non-kosmotropes might be more influenced by a shift from H_2_O to D_2_O. It is noteworthy that although Cl^-^ had this similarity with the strong chaotropes SCN^-^ and is usually classified as a weak chaotrope, its effect on the CST of both polymers was rather that of a kosmotropes, i.e., a reduction in CST (Figure 4). 

**Figure 7:**
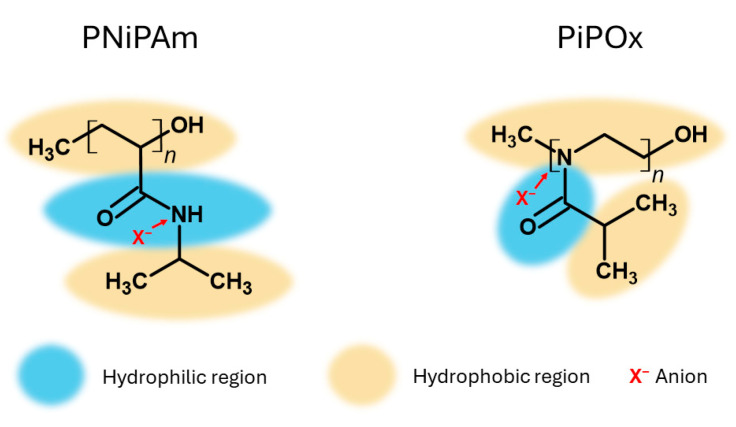
PNiPAm and PiPOx chemical structures with their polar and apolar regions marked. Yellow indicates exposed apolar moieties, blue polar moieties, and X^−^ anions directly binding to the amide group.

Cremer et al. similarly argue that chaotropes can stabilize amide side groups of PNiPAm and increase polymer solubility [15]. A schematic illustration of the proposed mechanism is shown in Figure 7. On the one hand, anions can affect the hydrogen bonding of hydrophilic structures (shown in blue in Figure 7) by polarizing the associated water molecules. They can additionally increase the interfacial tension between the hydrated structures and the cavity surrounding the apolar backbone and isopropyl side chains (indicated in yellow in Figure 7). These two effects could be responsible for destabilizing the polymers, therefore decreasing the CST. Additionally, the anions might bind directly to the amide groups, which should increase the CST.

Related to these observations, the solubility transition in the presence of chaotropic Hofmeister series salts, $SCN^{-}$ and $Cl^{-}$, seems to include at least two solvation transitions following upon each other for PiPOx in H_2_O (Figure 5). Remarkably, only one peak is observed in D_2_O for the same sample. It makes determining the CST using DSC by the peak *C*_p_ value ambiguous in H_2_O, but it also could elucidate additional features of the solvation transition. Tentatively, different parts of the polymer chains become dehydrated at different temperatures, reflecting preferential interactions of the anion with parts of the polymer. This could lead to significant differences between the CST determined by turbidity or DLS measurements and those determined by DSC. Unlike PNiPAm, PiPOx has an amide group in its backbone. Stronger hydration of ${\mathrm{SCN}}^{-}$ in water than in D_2_O may allow the anion to stabilize the polymer backbone longer, leading to dehydration of the side chains first, which might cause the multiple transitions for PiPOx samples with KSCN (or KCl) solved in H_2_O as well as the higher CST than PNiPAm. 

Curiously, polymer concentration greatly influenced the transition enthalpy of PNiPAm in D_2_O. The influence of polymer concentration on the CST is well known and was observed for both polymers and solvents in our study, but this effect on the transition enthalpy stands out and could not be fully explained [43,45]. Further, the observations of a two-times-higher enthalpy of the transition in D_2_O than H_2_O, which strongly decreased at a high polymer concentration, highlights that the intricate balance of several parameters of the investigated system, like intramolecular interactions and the breaking of intermolecular hydrogen bonds, must be considered if one wants to determine CST in a specific environment, in particular in D_2_O. 

Our observation of multiple transitions affected by the polarity of the solvent and the presence of Hofmeister anions requires further investigation of the molecular weight dependence of these features and whether they respond to different sidechain modifications. Previous publications have shown that the CST is decreased with increasing molecular weight for PNiPAm and PiPOx [41,46] and that sidechain modifications and polymer morphology, e.g., grafting, greatly influence the CST and internal coil and coil–coil desolvation transitions [20,47,48]. 

Finally, in this work, we focused on differences in the solubility transitions in H_2_O and D_2_O, respectively. One could presume that mixtures of H_2_O and D_2_O would influence the solubility according to the rule of mixtures, i.e., by a simple interpolation between the responses in each individual solvent. Although such behaviors are often observed for solvent mixtures, the local activity of ions and solvents on different parts of the polymer chain and side groups indicated by our data suggest that this assumption warrants further investigation.

## 4. Materials and Methods

### 4.1. Polymer Synthesis

#### 4.1.1. 2-Isopropyl-2-oxazoline

The following synthesis of the 2-isopropyl-2-oxazoline (iPOx) monomer adapts the Witte-Seeliger cyclocondensation described in Schroffenegger et al. [20,49,50].

Synthesis was conducted by mixing 45 mL (or 1 equivalent) of 2-methylpropanenitrile (Sigma-Aldrich, St. Louis, MI, USA) with 36 mL (1.2 eq.) ethanolamine (Sigma-Aldrich, St. Louis, MI, USA). Zinc acetate dihydrate (Sigma-Aldrich, St. Louis, MI, USA) was added to catalyze the reaction (2.2038 g or 0.02 eq.). Heated to 130 °C in an oil bath and equipped with a reflux cooler, the solution was left to react in an inert atmosphere for one day. Formed ammonia was absorbed in hydrochloric acid. The product was dissolved in dichloromethane (DCM, Sigma-Aldrich, St. Louis, MI, USA) and extracted with MQ-H_2_O until the pH of the aqueous phase was neutral. The organic phase was dried with sodium sulfate (Sigma-Aldrich, St. Louis, MI, USA). The solid was removed by filtration, and the product was separated from the solvent by vacuum distillation. After adding calcium hydride (Sigma-Aldrich, St. Louis, MI, USA), the solution was stirred under inert conditions to remove residual water. Finally, the product was purified using distillation with a Vigreux column at 50 °C and 50 mbar.

#### 4.1.2. Poly(2-isopropyl-2-oxazoline)

Polymerization of 2-Isopropyl-2-oxazoline followed Schroffenegger et al., who describe a cationic ring-opening polymerization (CROP) [20]. Initially, 3 mL of the monomer and 9 mL *N,N*-dimethylacetamide anhydrous (DMA, Sigma-Aldrich, St. Louis, MI, USA) were mixed with 38 µL methyl-*p*-tosylate (Sigma-Aldrich, St. Louis, MI, USA) and left to react at 90 °C overnight. The reaction was quenched with sodium hydroxide and stirred for three hours at 50 °C, then at room temperature until the next day. The product was obtained by stepwise precipitation of the reaction mixture in approximately 25 mL of a 1:1 mixture of *n*-hexane (Sigma-Aldrich, St. Louis, MI, USA) and diethyl ether (Carl Roth, Karlsruhe, Germany). The precipitate was then dissolved in DCM and purified by distillation.

Degree of polymerization (DP): 186; molecular weight (*M*_W_): 21 kDa; polydispersity index (PDI): 1.038; yield: 2.68 g, 94%; ^1^H-NMR *δ*_H_(300 MHz, CDCl_3_, ppm) 3.46 (d, 2H), 3.00 (s, 3H), 2.77 (d, 1H), 1.10 (s, 6H); ^13^C NMR *δ*_C_(75 MHz, CDCl_3_, ppm) 178, 46, 44, 30, 20.

#### 4.1.3. Poly(N-isopropylacrylamide)

This synthesis, described in Kurzhals et al., is an acid-terminated atomic transfer radical polymerization (ATRP) [51]. First, the monomer *N*-isopropyl acrylamide (NiPAm, Sigma-Aldrich, St. Louis, MI, USA) is recrystallized by dissolving 2 g NiPAm in 25 mL toluene (Carl Roth) and 35 mL *n*-hexane and storing it at −20 °C overnight. The precipitate was then filtered and rinsed with *n*-hexane, followed by vacuum distillation to remove the solvent.

NiPAm (0.8 g), copper(II) bromide (1.6 mg, Sigma-Aldrich, St. Louis, MI, USA), copper(I) bromide (10.3 mg, Sigma-Aldrich, St. Louis, MI, USA), and 2-bromo-2-methylpropionic acid (13.4 mg, Sigma-Aldrich, St. Louis, MI, USA) were dissolved in 10% methanol (Carl Roth, Karlsruhe, Germany) and purged with nitrogen while being cooled in an ice bath. After 20 min, 40 µL tris[2-(dimethylamino)ethyl]amine (Sigma-Aldrich, St. Louis, MI, USA) in 1 mL MQ-H_2_O was added to start the reaction, which continued for 24 h at 4 °C in an inert atmosphere. The polymerization was stopped by opening the flask to air, and the product separated from the reaction mixture by heating it to 50 °C to let the polymer precipitate. The supernatant was discarded. The gel-like, blue-colored polymer was dissolved in approximately 5 mL of tetrahydrofuran anhydrous (THF, Sigma-Aldrich, St. Louis, MI, USA) before reprecipitating in approximately 15 mL of cooled diethyl ether. The solid was dried in an evacuated desiccator. The polymer was solved again in Milli-Q H_2_O to remove impurities and dialyzed for four days; the water was changed five times. Finally, the polymer was then dried by lyophilization.

DP: 188; *M*_W_: 21 kDa; PDI: 1.049; yield: 0.62 g, 77%; ^1^H-NMR *δ*_H_(300 MHz, CDCl_3_, ppm) 4.02 (s, 1H), 2.14 (s, 1H), 1.66 (d, 2H), 1.15 (d, 6H); ^13^C NMR *δ*_C_(75 MHz, CDCl_3_, ppm) 174, 42, 41, 36, 23.

### 4.2. Polymer Characterization

The molecular weight (*M*_W_) and the polydispersity index (PDI) of PNiPAm and PiPOx were determined with gel permeation chromatography (GPC) based on their R_G_ against a standard of polystyrene in *N*,*N*-dimethylformamide (DMF) using a Malvern Viscotek GPC max and OmniSEC 5.12 software (Malvern Panalytical, Malvern, UK) (see Figures S6 and S10). For the measurement, 3.5 mg polymer was dissolved in 1 mL DMF containing 0.05 mol L^−1^ lithium bromide (Sigma-Aldrich, St. Louis, MI, USA). Then, 50 µL of sample was measured at a flow rate of 9.5 mL min^−1^ at 60 °C.

Successful synthesis in terms of correct chemical structure was investigated with nuclear magnetic resonance (^1^H NMR and ^13^C NMR) with a BRUKER AV III 300 MHz spectrometer (Bruker Austria GmbH, Vienna, Austria). A total of 50 mg mL^−1^ of either polymer was used to record the respective spectra in deuterated trichloromethane (Sigma-Aldrich, St. Louis, MI, USA). The chemical shifts (*δ* in ppm) were referenced to tetramethylsilane (TMS, Sigma-Aldrich, St. Louis, MI, USA); see Figures S3 and S4.

Infrared spectroscopy was performed with an FT-IR ATR spectrometer (Vertex 70, Bruker Austria GmbH, Vienna, Austria). Between 3000 cm^−1^ and 2800 cm^−1^, the PNiPAm spectrum shows the C-H stretching region. Between 1700 and 1300 cm^−1^, the fingerprint region, including the amide region with two strong peaks at approximately 1631 cm^−1^ and 1539 cm^−1^, is found. Peaks of the C-H deformation region are visible at 1462 cm^−1^ and 1388 cm^−1^. The PiPOx FT-IR spectrum also shows the C-H stretching region between 3000 cm^−1^ and 2800 cm^−1^. Similarly, a strong peak at ~1620 cm^−1^ is attributed to the amide group. Between 1473 cm^−1^ and 1363 cm^−1^, the C-H deformation mode is found. The respective spectra are shown in Figures S5 and S9.

The values for molecular weight and polydispersity obtained with GPC are collected in Table S5. They confirm that both polymers are of comparable size and low polydispersity. ^1^H NMR and ^13^C NMR spectra reveal that both polymers have the desired structure. The spectra are presented in Figures S3 and S4 for PNiPAm and Figures S7 and S8 for PiPOx, respectively, and the chemical shifts are provided above. The ^1^H NMR spectra show trace amounts of DCM from the synthesis. Its influence on the polymers’ thermal response is expected to be marginal. Importantly, the trace amounts of DCM will be the same in D_2_O and H_2_O samples and scale stoichiometrically with the polymer concentration in all samples. Hence, all relative comparisons are unaffected.

### 4.3. Determination of the Critical Solution Temperature and Transition Enthalpies

Solutions of PNiPAm and PiPOx in three different concentrations were prepared in H_2_O and D_2_O (0.1 mg mL^−1^, 1 mg mL^−1^, and 10 mg mL^−1^). Additionally, 1 mg mL^−1^ solutions of each polymer were prepared in solvent containing one of four potassium salts in different concentrations (160 mM, 10 mM, or 1 mM of $K_{2}{\mathrm{SO}}_{4}$ (≥99.0%, Sigma-Aldrich, St. Louis, MI, USA), $K_{2}{\mathrm{HPO}}_{4}$ (≥99%, Riedel-de Haën, Seelze, Germany), KCl (>99.5%, Fluka BioChemika, Ronkonkoma, NY, USA) and KSCN (≥98.0%, Merck, Rahway, NJ, USA), respectively). DLS size measurements were recorded with a step size of 1 kelvin and an equilibration time of 60 s. Close to the expected CST, increments were reduced to 0.2 K, and equilibration time after each step was increased to 120 s for higher precision. At each point, three individual measurements were recorded with an automatically set number of runs and then averaged. All samples were measured with a Zetasizer Nano ZS (Malvern Panalytical, Malvern, UK) in backscatter mode. CST values were extracted at the initial increase in the recorded scattering patterns (see also Figure S1). For DSC experiments, 325 µL of each sample was scanned from 20 °C to 80 °C at a rate of 1 °C per minute with a MicroCal PEAQ-DSC Automated (Malvern Panalytical, Malvern, UK). Before starting the measurements, the system was thermally equilibrated for 5 min, and 3 purge refills of the autosampler were performed between samples. CST values were extracted from the recorded thermograms at the transition peak maximum.

Transition enthalpy values are taken directly from the MicroCal PEAQ-DSC software 1.53 and obtained by integrating the thermograms after buffer subtraction and baseline correction according to Kirchoff’s law:

(1) $$\Delta H=\int_{T_{1}}^{T_{2}}C_{P}dT,$$

where ΔH is the change in enthalpy, $C_{P}$ the heat capacity at constant pressure, and $T_{1}$ and $T_{2}$ the temperatures before and after the transition, respectively.

## 5. Conclusions

We conclude that the thermoresponsive properties of polymers quantitatively differ in D_2_O and H_2_O, but the absolute differences depend on the polymer and solution conditions. Predominantly, D_2_O decreased the CST in the pure solvent compared to H_2_O. The observed effects of changing from H_2_O to D_2_O were smaller than those of other commonly varied parameters, such as polymer concentration, molecular weight, copolymerization, or the presence of physiological concentrations of Hofmeister series ions [52,53,54]. Still, they are far from negligible given that they amount to shifts of several degrees of the CST, which could increase by the addition of Hofmeister series ions, in particular the chaotropes we tested. Our results strongly imply that the effects of different environmental parameters, i.e., anion species and concentration, are not additive with a change from H_2_O to D_2_O. For instance, the dehydration of different molecular groups in a polymer can be differently affected, as well as their interactions with Hofmeister series anions, which, in turn, influence the overall thermoresponsive behavior. This was tentatively observed for the influence of amine polar bonds and chaotropic anions. These polymer-internal changes to the transition could be especially important if more complicated polymer morphologies, such as dendritic polymers or grafted polymers, are studied. Our findings regarding multiple transitions within PiPOx in the presence of chaotropes indicate the need to extend our study to a larger set of polymer side groups, molecular weights, chain ends, and topologies relevant to applications. The strong influence of physiological concentrations of Cl^−^, affecting the solvation transition in D_2_O in a non-additive way, highlights that care must be taken to compare results obtained by techniques relying on D_2_O and H_2_O, respectively, for conclusions regarding designs for biomedical applications.

## Figures and Tables

**Table 1:** DSC-derived transition enthalpies per monomer unit for differently concentrated PNiPAm and PiPOx dispersions (21 kDa) prepared in H_2_O and D_2_O.

**PNiPAm**	**Δ*H*_monomer_ [J mol^−1^]**	
***c* [mg mL^−1^]**	**H_2_O**	**D_2_O**
0.1	4178	11,838
1	5568	11,459
10	5335	5676
**PiPOx**	**Δ*H*_monomer_ [J mol^−1^]**	
***c* [mg mL^−1^]**	**H_2_O**	**D_2_O**
0.1	3708	4865
1	5324	5259
10	4535	5189

## Data Availability

Data will be made available on request by the authors.

## Supplementary Materials

The following supporting information can be downloaded at: https://www.mdpi.com/article/10.3390/ijms25147734/s1.

## References

[1] Karati D. (2023). A Concise Review on Bio-Responsive Polymers in Targeted Drug Delivery System. Polym. Bull..

[2] Han J., Sheng T., Zhang Y., Cheng H., Gao J., Yu J., Gu Z. (2023). Bioresponsive Immunotherapeutic Materials. Adv. Mater..

[3] Barhoum A., Sadak O., Ramirez I.A., Iverson N. (2023). Stimuli-Bioresponsive Hydrogels as New Generation Materials for Implantable, Wearable, and Disposable Biosensors for Medical Diagnostics: Principles, Opportunities, and Challenges. Adv. Colloid Interface Sci..

[4] Heskins M., Guillet J.E. (1968). Solution Properties of Poly(N-Isopropylacrylamide). J. Macromol. Sci. Part—Chem..

[5] Pasparakis G., Tsitsilianis C. (2020). LCST Polymers: Thermoresponsive Nanostructured Assemblies towards Bioapplications. Polymer.

[6] Kotsuchibashi Y. (2020). Recent Advances in Multi-Temperature-Responsive Polymeric Materials. Polym. J..

[7] Niemeyer C.M., Meyers R.A. (2006). Nanobiotechnology. Encyclopedia of Molecular Cell Biology and Molecular Medicine.

[8] Hogan K.J., Mikos A.G. (2020). Biodegradable Thermoresponsive Polymers: Applications in Drug Delivery and Tissue Engineering. Polymer.

[9] Castillo-Henríquez L., Castro-Alpízar J., Lopretti-Correa M., Vega-Baudrit J. (2021). Exploration of Bioengineered Scaffolds Composed of Thermo-Responsive Polymers for Drug Delivery in Wound Healing. Int. J. Mol. Sci..

[10] Lechuga-Islas V.D., Sánchez-Cerrillo D.M., Stumpf S., Guerrero-Santos R., Schubert U.S., Guerrero-Sánchez C. (2023). Thermo-Responsive Polymer Catalysts for Polyester Recycling Processes: Switching from Homogeneous Catalysis to Heterogeneous Separations. Polym. Chem..

[11] Moghaddam S.Z., Thormann E. (2016). Hofmeister Effect on Thermo-Responsive Poly(Propylene Oxide) in H2O and D2O. RSC Adv..

[12] Hofmeister F. (1888). Zur Lehre von der Wirkung der Salze: Dritte Mittheilung. Arch. Exp. Pathol. Pharmakol..

[13] Suwa K., Yamamoto K., Akashi M., Takano K., Tanaka N., Kunugi S. (1998). Effects of Salt on the Temperature and Pressure Responsive Properties of Poly(N -Vinylisobutyramide) Aqueous Solutions. Colloid Polym. Sci..

[14] Zhang Y., Cremer P.S. (2006). Interactions between Macromolecules and Ions: The Hofmeister Series. Curr. Opin. Chem. Biol..

[15] Zhang Y., Furyk S., Bergbreiter D.E., Cremer P.S. (2005). Specific Ion Effects on the Water Solubility of Macromolecules:  PNIPAM and the Hofmeister Series. J. Am. Chem. Soc..

[16] Moelbert S., Normand B., De Los Rios P. (2004). Kosmotropes and Chaotropes: Modelling Preferential Exclusion, Binding and Aggregate Stability. Biophys. Chem..

[17] Scheiner S., Čuma M. (1996). Relative Stability of Hydrogen and Deuterium Bonds. J. Am. Chem. Soc..

[18] Cho Y., Sagle L.B., Iimura S., Zhang Y., Kherb J., Chilkoti A., Scholtz J.M., Cremer P.S. (2009). Hydrogen Bonding of β-Turn Structure Is Stabilized in D2O. J. Am. Chem. Soc..

[19] Lopez M.M., Makhatadze G.I. (1998). Solvent Isotope Effect on Thermodynamics of Hydration. Biophys. Chem..

[20] Schroffenegger M., Zirbs R., Kurzhals S., Reimhult E. (2018). The Role of Chain Molecular Weight and Hofmeister Series Ions in Thermal Aggregation of Poly(2-Isopropyl-2-Oxazoline) Grafted Nanoparticles. Polymers.

[21] Halperin A., Kröger M., Winnik F.M. (2015). Poly(N-Isopropylacrylamide) Phase Diagrams: Fifty Years of Research. Angew. Chem. Int. Ed..

[22] Guan Y., Zhang Y. (2011). PNIPAM Microgels for Biomedical Applications: From Dispersed Particles to 3D Assemblies. Soft Matter.

[23] Tang L., Wang L., Yang X., Feng Y., Li Y., Feng W. (2021). Poly(N-Isopropylacrylamide)-Based Smart Hydrogels: Design, Properties and Applications. Prog. Mater. Sci..

[24] Okano T., Yamada N., Sakai H., Sakurai Y. (1993). A Novel Recovery System for Cultured Cells Using Plasma-Treated Polystyrene Dishes Grafted with Poly(N-Isopropylacrylamide). J. Biomed. Mater. Res..

[25] Doberenz F., Zeng K., Willems C., Zhang K., Groth T. (2020). Thermoresponsive Polymers and Their Biomedical Application in Tissue Engineering—A Review. J. Mater. Chem. B.

[26] Zhang Q., Weber C., Schubert U.S., Hoogenboom R. (2017). Thermoresponsive Polymers with Lower Critical Solution Temperature: From Fundamental Aspects and Measuring Techniques to Recommended Turbidimetry Conditions. Mater. Horiz..

[27] Hoogenboom R. (2009). Poly(2-Oxazoline)s: A Polymer Class with Numerous Potential Applications. Angew. Chem. Int. Ed..

[28] Weber C., Hoogenboom R., Schubert U.S. (2012). Temperature Responsive Bio-Compatible Polymers Based on Poly(Ethylene Oxide) and Poly(2-Oxazoline)s. Prog. Polym. Sci..

[29] Luxenhofer R., Sahay G., Schulz A., Alakhova D., Bronich T.K., Jordan R., Kabanov A.V. (2011). Structure-Property Relationship in Cytotoxicity and Cell Uptake of Poly(2-Oxazoline) Amphiphiles. J. Control. Release.

[30] Hoogenboom R., Schlaad H. (2017). Thermoresponsive Poly(2-Oxazoline)s, Polypeptoids, and Polypeptides. Polym. Chem..

[31] Shirota H., Endo N., Horie K. (1998). Volume Phase Transition of Polymer Gel in Water and Heavy Water. Chem. Phys..

[32] Kurzhals S., Schroffenegger M., Gal N., Zirbs R., Reimhult E. (2018). Influence of Grafted Block Copolymer Structure on Thermoresponsiveness of Superparamagnetic Core–Shell Nanoparticles. Biomacromolecules.

[33] Litt M.H., Hsieh B.R., Krieger I.M., Chen T.T., Lu H.L. (1987). Low Surface Energy Polymers and Surface-Active Block Polymers: II. Rigid Microporous Foams by Emulsion Polymerization. J. Colloid Interface Sci..

[34] Pérez-Fuentes L., Bastos-González D., Faraudo J., Drummond C. (2018). Effect of Organic and Inorganic Ions on the Lower Critical Solution Transition and Aggregation of PNIPAM. Soft Matter.

[35] Pham Q.-T., Yao Z.-H., Chang Y.-T., Wang F.-M., Chern C.-S. (2018). LCST Phase Transition Kinetics of Aqueous Poly(N-Isopropylacrylamide) Solution. J. Taiwan Inst. Chem. Eng..

[36] Schild H.G., Tirrell D.A. (1990). Microcalorimetric Detection of Lower Critical Solution Temperatures in Aqueous Polymer Solutions. J. Phys. Chem..

[37] Kunugi S., Tada T., Tanaka N., Yamamoto K., Akashi M. (2002). Microcalorimetric Study of Aqueous Solution of a Thermoresponsive Polymer, Poly(N-Vinylisobutyramide) (PNVIBA). Polym. J..

[38] Uyama H., Kobayashi S. (1992). A Novel Thermo-Sensitive Polymer. Poly(2-Iso-Propyl-2-Oxazoline). Chem. Lett..

[39] Pamies R., Zhu K., Kjøniksen A.-L., Nyström B. (2009). Thermal Response of Low Molecular Weight Poly-(N-Isopropylacrylamide) Polymers in Aqueous Solution. Polym. Bull..

[40] Wang X., Wu C. (1999). Light-Scattering Study of Coil-to-Globule Transition of a Poly(N-Isopropylacrylamide) Chain in Deuterated Water. Macromolecules.

[41] Diab C., Akiyama Y., Kataoka K., Winnik F.M. (2004). Microcalorimetric Study of the Temperature-Induced Phase Separation in Aqueous Solutions of Poly(2-Isopropyl-2-Oxazolines). Macromolecules.

[42] Qiu X., Koga T., Tanaka F., Winnik F.M. (2013). New Insights into the Effects of Molecular Weight and End Group on the Temperature-Induced Phase Transition of Poly(N-Isopropylacrylamide) in Water. Sci. China Chem..

[43] Zhao J., Hoogenboom R., Van Assche G., Van Mele B. (2010). Demixing and Remixing Kinetics of Poly(2-Isopropyl-2-Oxazoline) (PIPOZ) Aqueous Solutions Studied by Modulated Temperature Differential Scanning Calorimetry. Macromolecules.

[44] Swain C.G., Bader R.F. (1960). The Nature of the Structure Difference between Light and Heavy Water and the Origin of the Solvent Isotope Effect—I. Tetrahedron.

[45] Fehér B., Varga I., Pedersen J.S. (2022). Effect of Concentration and Ionic Strength on the Lower Critical Solution Temperature of Poly(N-Isopropylacrylamide) Investigated by Small-Angle X-Ray Scattering. Soft Mater..

[46] Furyk S., Zhang Y., Ortiz-Acosta D., Cremer P.S., Bergbreiter D.E. (2006). Effects of End Group Polarity and Molecular Weight on the Lower Critical Solution Temperature of Poly(N-Isopropylacrylamide). J. Polym. Sci. Part Polym. Chem..

[47] Jhon Y.K., Bhat R.R., Jeong C., Rojas O.J., Szleifer I., Genzer J. (2006). Salt-Induced Depression of Lower Critical Solution Temperature in a Surface-Grafted Neutral Thermoresponsive Polymer. Macromol. Rapid Commun..

[48] Schroffenegger M., Reimhult E. (2018). Thermoresponsive Core-Shell Nanoparticles: Does Core Size Matter?. Materials.

[49] Witte H., Seeliger W. (1974). Cyclische Imidsäureester aus Nitrilen und Aminoalkoholen. Justus Liebigs Ann. Chem..

[50] Monnery B.D., Shaunak S., Thanou M., Steinke J.H.G. (2015). Improved Synthesis of Linear Poly(Ethylenimine) via Low-Temperature Polymerization of 2-Isopropyl-2-Oxazoline in Chlorobenzene. Macromolecules.

[51] Kurzhals S., Zirbs R., Reimhult E. (2015). Synthesis and Magneto-Thermal Actuation of Iron Oxide Core–PNIPAM Shell Nanoparticles. ACS Appl. Mater. Interfaces.

[52] Xu S., Trujillo F.J., Xu J., Boyer C., Corrigan N. (2021). Influence of Molecular Weight Distribution on the Thermoresponsive Transition of Poly(N-Isopropylacrylamide). Macromol. Rapid Commun..

[53] Smith A.A., Maikawa C.L., Hernandez H.L., Appel E.A. (2021). Controlling Properties of Thermogels by Tuning Critical Solution Behaviour of Ternary Copolymers. Polym. Chem..

[54] Otulakowski Ł., Kasprów M., Strzelecka A., Dworak A., Trzebicka B. (2021). Thermal Behaviour of Common Thermoresponsive Polymers in Phosphate Buffer and in Its Salt Solutions. Polymers.

