# Supplementary material for: Effect of Solvent Properties on the Critical Solution Temperature of Thermoresponsive Polymers
Source: Int J Mol Sci. 2024 Jul 15;25(14):7734. doi: 10.3390/ijms25147734 (PMC11277098; doi:10.3390/ijms25147734)
Supplement: Supplementary file 1 [file ijms-25-07734-s001.zip › ijms-3022354-supplementary.pdf]

Supplementary information

Effect of Solvent Properties on the Critical Solution Temperature of Thermoresponsive Polymers

Konstantin Nikolaus Beitl[1] and Erik Reimhult[1,*]

[1]Institute of Colloid and Biointerface Science, Department of Bionanosciences, BOKU University, Muthgasse 11, A-1190 Vienna, Austria

*Correspondence: erik.reimhult@boku.ac.at

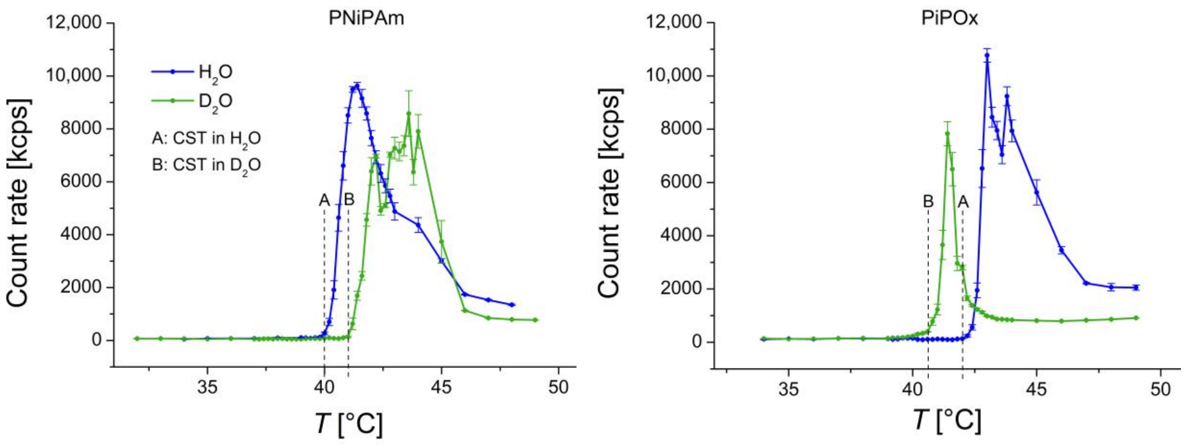

(**a**) (**b**)

Figure S1. Extraction of the Critical Solution Temperature (CST) from DLS measurements exemplarily shown for (a) PNiPAm (21 kDa; 1 mg ml^{-1}) and (b) PiPOx (21 kDa; 1 mg ml^{-1}) in H_2O (blue) and D_2O (green). At each temperature point, three measurements of a minimum ten runs each were conducted, and the average plotted. Lines A and B indicate where the CST was extracted from the recorded curves.

Table S1. Critical solution temperatures of differently concentrated PNiPAm and PiPOx (21 kDa) dispersions prepared in H_2O and D_2O measured with DLS and DSC, respectively.

**PNiPAm (DLS)**	**H_2O**	**D_2O**	**PNiPAm (DSC)**	**H_2O**	**D_2O**
c [mg ml^{-1}]	***T* [°C]**		*c* [mg ml^{-1}]	***T* [°C]**	
0.1	45.0	43.4	0.1	46.49	44.52
1	40.0	41.0	1	43.54	42.32
10	35.4	36.6	10	39.94	38.67
**PiPOx (DLS)**	**H_2O**	**D_2O**	**PiPOx (DSC)**	**H_2O**	**D_2O**
c [mg ml^{-1}]	***T* [°C]**		*c* [mg ml^{-1}]	***T* [°C]**	
0.1	45.0	43.8	0.1	46.39	45.27

1	42.0	40.6	1	43.29	42.57
10	38.6	37.8	10	40.27	39.55

Table S2. Comparison of onset and peak temperature values [°C] in DSC thermograms of PNiPAm and PiPOx dispersions (21 kDa polymers; 10^{-1} mg ml^{-1}, 10^{0} mg ml^{-1} and 10^{1} mg ml^{-1}) in H_2O and D_2O.

**PNiPAm**	**H_2O**			**D_2O**		
c [mg ml^{-1}]	T_{onset}	T_{peak}	**ΔT**	T_{onset}	T_{peak}	**ΔT**
0.1	43.94	46.49	**2.55**	39.27	44.52	**5.25**
1	41.34	43.54	**2.20**	40.04	42.32	**2.28**
10	37.83	39.94	**2.11**	38.11	38.67	**0.56**
**PiPOx**	**H_2O**			**D_2O**		
c [mg ml^{-1}]	T_{onset}	T_{peak}	**ΔT**	T_{onset}	T_{peak}	**ΔT**
0.1	43.08	46.39	**3.31**	40.58	45.27	**4.69**
1	41.3	43.29	**1.99**	40.63	42.57	**1.94**
10	39.19	40.27	**1.08**	38.58	39.55	**0.97**

Table S3. Critical solution temperatures of PNiPAm and PiPOx (21 kDa; 1 mg ml^{-1}) in Hofmeister series salt solutions prepared in H_2O and D_2O from 1 mM to physiological ionic strength measured with DLS and DSC, respectively.

**PNiPAm**	**1 mM salts**		**10 mM salts**		**160 mM salts**	
**DLS**	T_{H2O}	T_{D2O}	T_{H2O}	T_{D2O}	T_{H2O}	T_{D2O}
No salt	40.2 (0 mM)	41.0 (0 mM)	-	-	-	-
K_2SO_4	40.4	40.2	39.4	39.4	31.2	32.0
K_2HPO_4	40.6	40.4	39.8	39.8	28.8	31.8
KCl	40.4	40.4	40.2	40.2	37.0	36.8
KSCN	40.4	40.4	40.4	40.4	39.2	42.6
**PNiPAm**	**1 mM salts**		**10 mM salts**		**160 mM salts**	
**DSC**	T_{H2O}	T_{D2O}	T_{H2O}	T_{D2O}	T_{H2O}	T_{D2O}
No salt	43.54 (0 mM)	42.32 (0 mM)	-	-	-	-
K_2SO_4	43.1	43.61	42.11	42.01	34.42	35.16
K_2HPO_4	43.9	44.08	43.75	43.71	34.57	35.73
KCl	42.83	43.6	42.68	42.32	39.84	40.45
KSCN	43.00	43.73	42.73	42.93	44.29	44.87
**PiPOx**	**1 mM salts**		**10 mM salts**		**160 mM salts**	
**DLS**	T_{H2O}	T_{D2O}	T_{H2O}	T_{D2O}	T_{H2O}	T_{D2O}
No salt	42.2 (0 mM)	40.2 (0 mM)	-	-	-	-
K_2SO_4	41.8	40.8	40.6	39.8	32.0	31.4
K_2HPO_4	41.2	40.2	40.4	39.4	31.4	31.2
KCl	41.4	40.6	41.2	39.6	39.2	35.6
KSCN	41.2	40.0	40.8	41.2	52.8	51.8
**PiPOx**	**1 mM salts**		**10 mM salts**		**160 mM salts**	
**DSC**	T_{H2O}	T_{D2O}	T_{H2O}	T_{D2O}	T_{H2O}	T_{D2O}
No salt	43.29 (0 mM)	42.57 (0 mM)	-	-	-	-
K_2SO_4	43.45	42.61	43.22	42.09	35.39	35.41
K_2HPO_4	43.12	42.36	42.32	41.1	34.35	34.23
KCl	43.51	42.65	43.53	42.06	41.9	41.69
KSCN	43.03	42.15	43.07	42.98	52.75	53.35

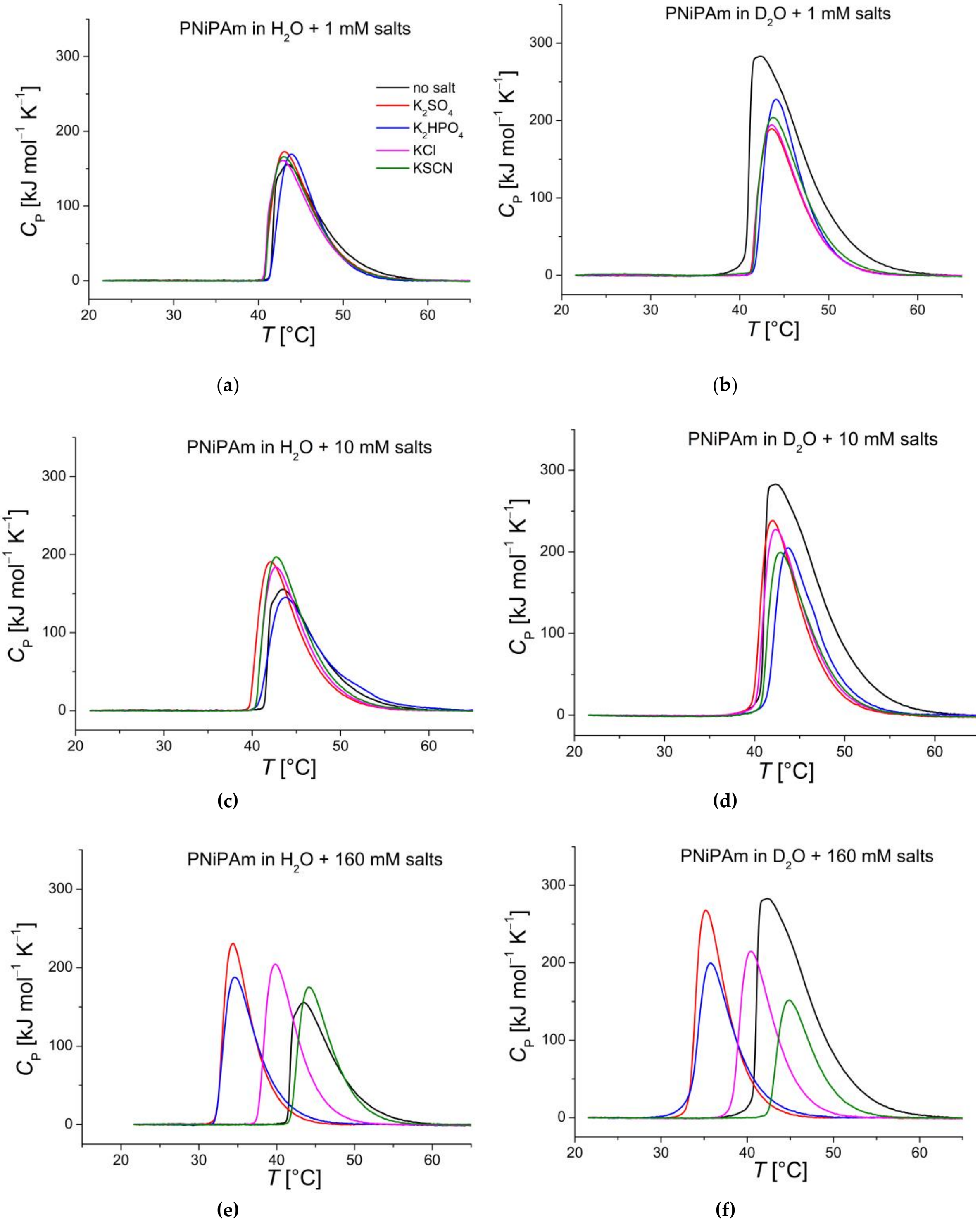

Figure S2. DSC-recorded thermograms of PNiPAm (21 kDa; 1 mg ml^{-1}) in H_2O and D_2O. Red: K_2SO_4, blue: K_2HPO_4, pink: KCl, green: KSCN; polymer dispersed in solutions of (a) 1 mM salts in H_2O; (b) 1 mM salts in D_2O; (c) 10 mM salts in H_2O; (d) 10 mM salts in D_2O; (e) 160 mM salts in H_2O and (f) 160 mM salts in D_2O.

Table S4. DSC-derived transition enthalpies per monomer unit of PNiPAm and PiPOx (21kDa) in differently concentrated salt solutions prepared in H_2O and D_2O.

**PNiPAm**	$\Delta H_{monomer}$ [J mol^{-1}]						
H_2O	**1 mM salt**	**10 mM salt**	**160 mM salt**	D_2O	**1 mM salt**	**10 mM salt**	**160 mM salt**
No salt	5568 (0 mM)	-	-	No salt	11459 (0 mM)	-	-
K_2SO_4	5676	5946	5838	K_2SO_4	5892	7135	6703
K_2HPO_4	4968	5730	5514	K_2HPO_4	6432	6324	5838
KCl	5375	5676	5730	KCl	5946	6973	5892
KSCN	5514	6270	5195	KSCN	6649	6162	4243
**PiPOx**	$\Delta H_{monomer}$ [J mol^{-1}]						
H_2O	**1 mM salts**	**10 mM salts**	**160 mM salts**	D_2O	**1 mM salts**	**10 mM salts**	**160 mM salts**
No salt	4719 (0 mM)	-	-	No salt	4470 (0 mM)	-	-
K_2SO_4	5189	4227	5568	K_2SO_4	5368	4222	6703
K_2HPO_4	5286	3357	5027	K_2HPO_4	5011	5514	6162
KCl	4859	4541	9135	KCl	5676	5459	9514
KSCN	4730	7838	15459	KSCN	5784	12865	18000

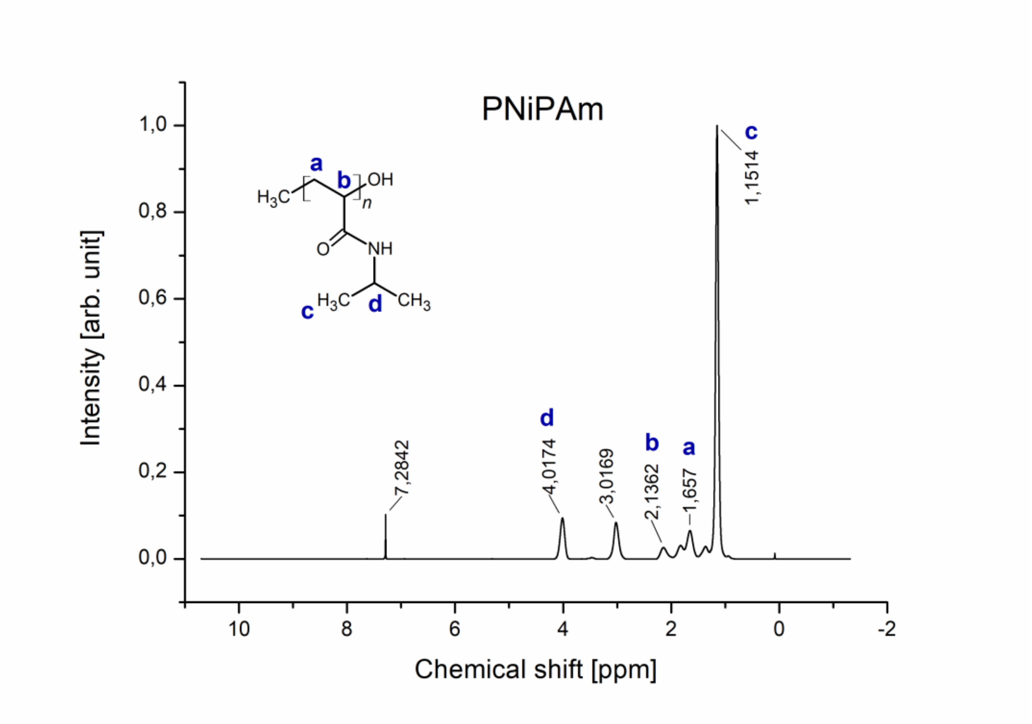

Figure S3. ^{1}H NMR spectrum for PNiPAm in $CDCl_3$ at 300 MHz. Normalised intensity is plotted against chemical shifts relative to TMS (0 ppm). Structural components and corresponding peaks are marked with letters **a** to **d**. PNiPAm: δH(300 MHz, $CDCl_3$, ppm) 4.02 (s, 1H), 2.14 (s, 1H), 1.66 (d, 2H), 1.15 (d, 6H)

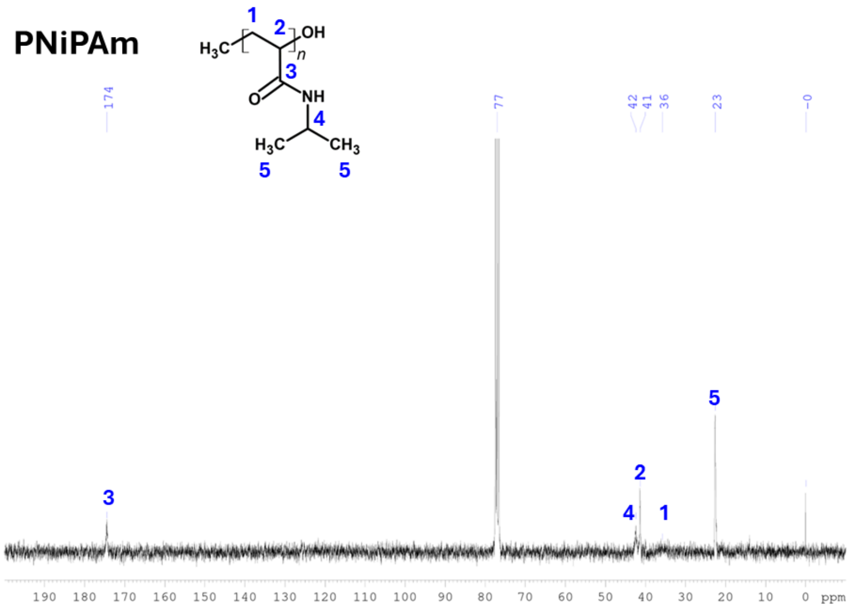

Figure S4. ^{13}C NMR spectrum for PNiPAm in $CDCl_3$ at 75 MHz. Normalised intensity is plotted against chemical shifts relative to TMS (0 ppm). Structural components and corresponding peaks are marked with letters **1** to **5**. PNiPAm: ^{13}C NMR δ_C(75 MHz, $CDCl_3$, ppm) 174, 42, 41, 36, 23)

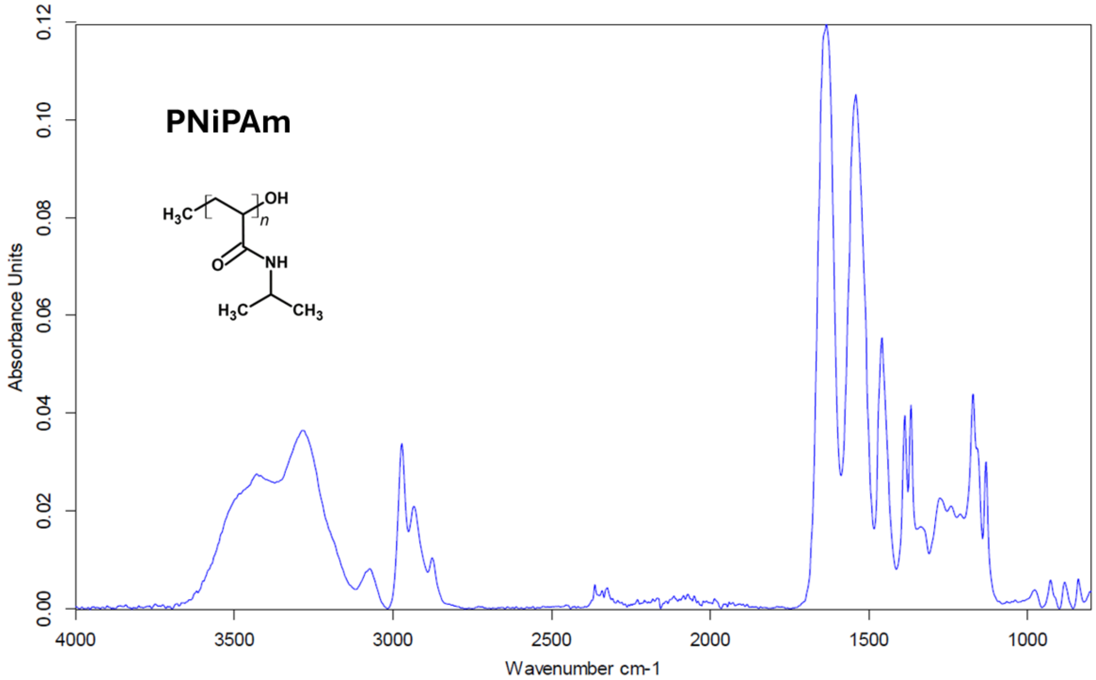

Figure S5. PNiPAm FT-IR spectrum.

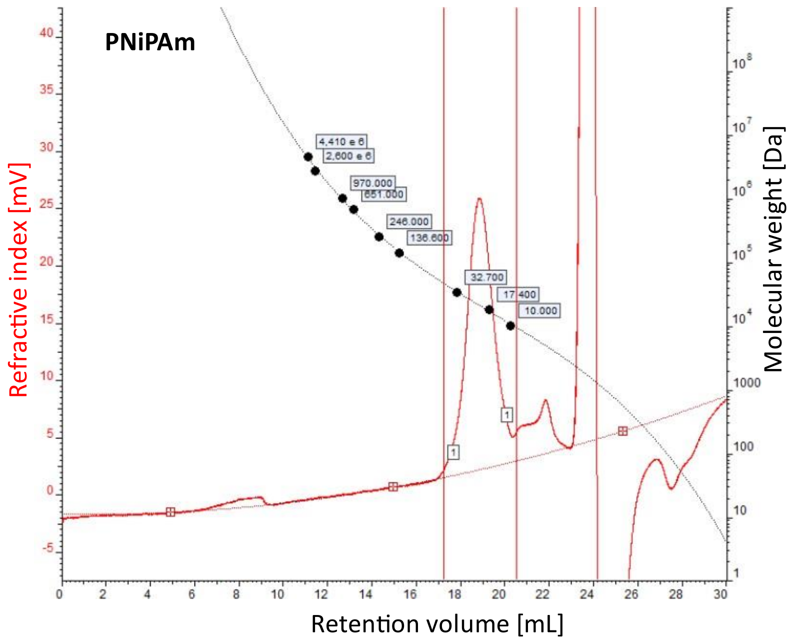

Figure S6. Gel permeation chromatography of PNiPAm based on its R$_G$ against a standard of polystyrene in *N*,*N*-dimethylformamide (DMF). 3.5 mg polymer were dissolved in 1 ml DMF containing 0.05 mol l^{-1} lithium bromide. 50 µl of sample were measured at a flow rate of 9.5 ml min^{-1} at 60 °C.

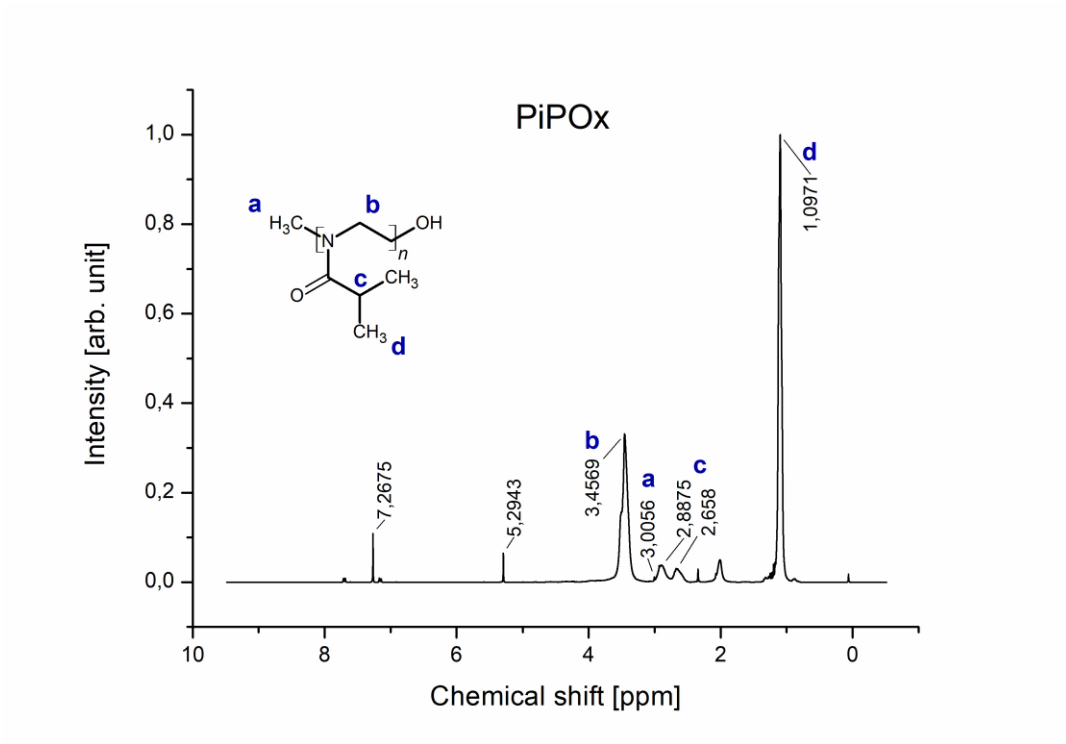

Figure S7. ^{1}H NMR spectrum for PiPOx in $CDCl_3$ at 300 MHz. Normalised intensity is plotted against chemical shifts relative to TMS (0 ppm). Structural components and corresponding peaks are marked with letters **a** to **d**. PiPOx: δ_H(300 MHz, $CDCl_3$, ppm) 3.46 (d, 2H), 3.00 (s, 3H), 2.77 (d, 1H), 1.10 (s, 6H).

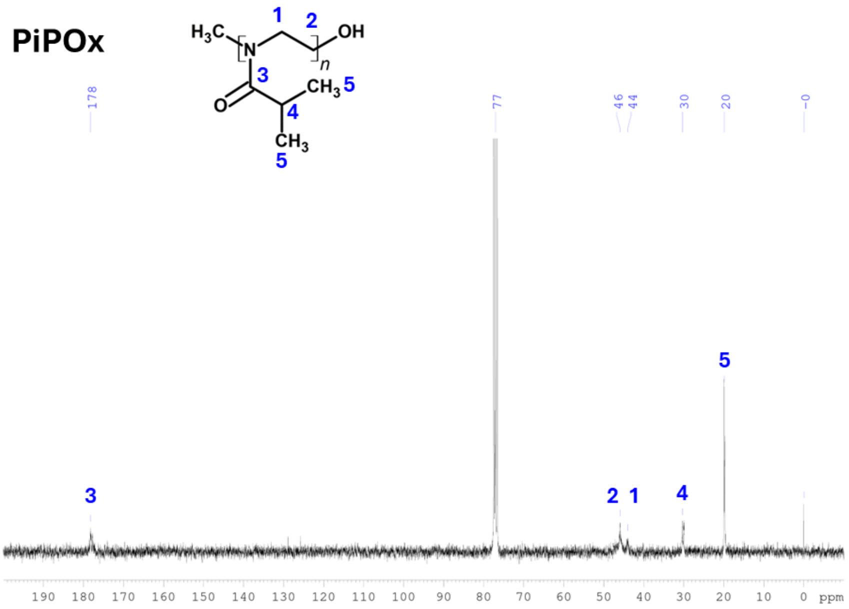

Figure S8. ^{13}C NMR spectrum for PiPOx in $CDCl_3$ at 75 MHz. Normalised intensity is plotted against chemical shifts relative to TMS (0 ppm). Structural components and corresponding peaks are marked with numbers **1** to **5**. PiPOx: ^{13}C NMR δ_C(75 MHz, $CDCl_3$, ppm) 178, 46, 44, 30, 20).

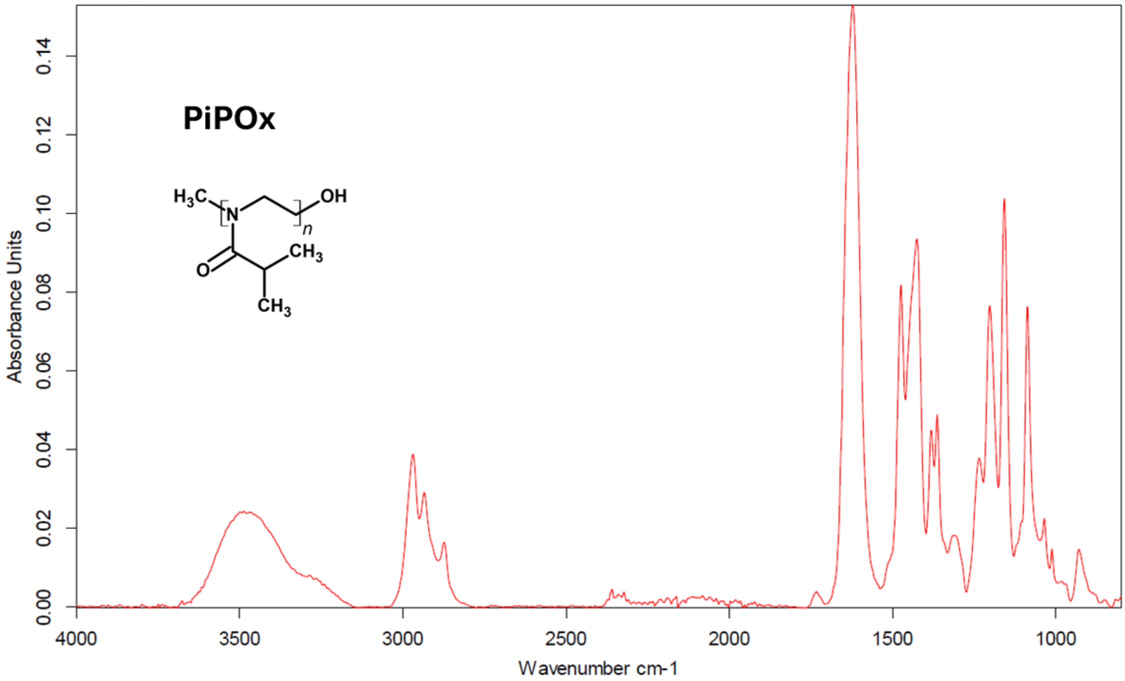

Figure S9. PiPOx FT-IR spectrum.

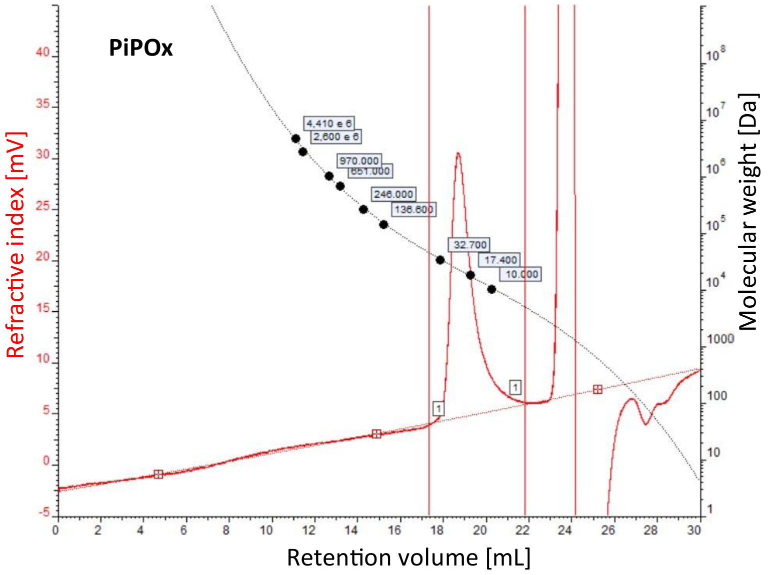

Figure S10. Gel permeation chromatography of PiPOx based on its R_G against a standard of polystyrene in *N*,*N*-dimethylformamide (DMF). 3.5 mg polymer were dissolved in 1 ml DMF containing 0.05 mol l^{-1} lithium bromide. 50 µl of sample were measured at a flow rate of 9.5 ml min^{-1} at 60 °C.

Table S5. Polymer weight and polydispersity derived from GPC.

Polymer	**PNiPAm**	**PiPOx**
Linear Formula	$CH_3(C_6H_{11}NO)_nOH$	$CH_3(C_6H_{11}NO)_nOH$
M_w [kDa]	21.368	21.119
M_n [kDa]	20.379	20.341
PDI	1.049	1.038

$$\mathrm{PDI} = \frac{M_\mathrm{w}}{M_n} \quad \text{Eq. S1}$$
